# Comparison of the heterochromatin and telomeric sequences distribuition in chromosomes of 11 species of Amazonian marsupials (Didelphimorphia; Didelphidae)

**DOI:** 10.1590/1678-4685-GMB-2019-0357

**Published:** 2020-05-11

**Authors:** Carlos Eduardo Faresin e Silva, Érica Martinha Silva de Souza, Eduardo Schmidt Eler, Maria Nazareth Ferreira da Silva, Eliana Feldberg

**Affiliations:** 1 Instituto Nacional de Pesquisas da Amazônia, Laboratório de Genética Animal, Manaus, AM, Brazil; 2 Instituto Nacional de Pesquisas da Amazônia, Coleção de Mamíferos, Manaus, AM, Brazil; 3 Universidade Anhembi Morumbi, Escola de Ciências da Saúde, São José dos Campos, SP, Brazil

**Keywords:** C-banding, ITS, repetitive sequences, FISH

## Abstract

In recent decades the diploid numbers recorded in the New World marsupials have been widely discussed in the context of the processes of karyotype evolution in these mammals. While Interstitial Telomeric Sequences (ITS) have long been interpreted as remnants of chromosomal fusion, the biological role of these features, together with their intraspecific variation, has raised a number of questions. In the present study, we analyzed the karyotype of 11 species of Amazonian didelphids, comparing the distribution of the heterochromatin with that of the telomeric signals, and found that, in six species, the ITS coincided with the blocks of heterochromatin. While ITS were found in the X chromosomes of all *Marmosa murina* individuals, they were variable in all the other species, representing a specific character of each lineage. Our results support the conclusion that ITS may not always be a consequence of chromosomal rearrangements, and that the mechanisms that produce them are still unclear.

The most common diploid numbers among marsupials are 2n = 14 and 2n = 22, in both New World and Australian species (Sharman, 1961, 1982; Biggers *et al.*, 1965; Hayman and Martin, 1974; Rofe and Hayman, 1985; Hayman, 1990). There is also a high degree of homeology among these species in the chromosome arms (De Leo *et al.*, 1999; Rens *et al.*, 1999). Based on these chromosomal homeologies, the marsupials are highly conserved, with the principal chromosomal rearrangements occurring through fusions or fissions (Reig *et al.*, 1977; O'Neill *et al.*, 1999; Rens *et al.*, 2003).

Comparative phylogenetic analyses of karyotypes have shown that the most common marsupial karyotypes have arisen more than once in different lineages, and according to Westerman *et al.* (2010), are invariably derived from the 2n = 14 through fissions. In this context, the mapping of telomere sequences has provided an important complementary perspective for the understanding of the karyotypic evolution of these organisms (Svartman and Vianna-Morgante, 1998).

Telomeric sequences are highly conserved and they protect the cohesive extremities of the linear eukaryote chromosome, preventing their interaction with other chromatids and the action of DNases (McClintock, 1941; Meyne *et al.*, 1989; Blackburn, 1991). However, these sequences have been found not only in telomeric regions, but also in interstitial (ITS) or centromeric regions, where they are interpreted as being the result of damage to the DNA, or the action of dispersed repetitions and chromosomal reorganization (Azzalin *et al.*, 2001; Ruiz-Herrera *et al.*, 2008; Sánchez *et al.*, 2010). Whatever their origin, the ITSs may be fixed in the genome, operating as markers for the analysis of evolutionary processes (Nergadze *et al.*, 2004).

In the Didelphidae, these sequences have been detected in centromeric regions, in species with both 2n = 14 and 2n = 18 chromosomes, which indicates that they are remnants of fusions from a 2n = 22 karyotype (Svartman and Vianna-Morgante, 1998; Carvalho and Mattevi, 2000). However, Pagnozzi *et al.* (2000, 2002) analyzed these markers in didelphid species, and found that the ITSs overlap with the heterochromatin and varied in number intraspecifically, indicating that they were not necessarily the result of recent fusions. Metcalfe *et al.* (2004) also concluded that not all the ITSs found in Australian marsupials of the family Macropodidae are evidence of fusions, except when they are the principal components of the heterochromatin. Subsequently, Svartman (2009) concluded that condensation of the chromosomes may make the mapping of telomeric sequences relatively imprecise, given that they are visualized in the region of the pericentromeric heterochromatin, when in fact these sequences are not located within the heterochromatin.

Comparative cytogenetic analyses are available for a large number of Australian marsupials, supporting inferences on their evolutionary patterns and the origin of their karyotypes. However, much fewer data are available for the New World, particularly for Amazonian species (Nagamachi *et al.*, 2015). The present study compared the position of the ITS and heterochromatin blocks in 11 didelphid species, all sampled in the Brazilian Amazon region, with the aim of shading light on the evolutionary patterns of this group.

We analyzed 11 Amazonian marsupial species, representing eight of the 12 genera, collected at 12 distinct localities (Figure 1, Table 1). We analyzed the same group of individuals as Silva *et al.* (2017). The individuals were identified by one of us (MNFS), using morphological and chromosomal characters. All voucher specimens were deposited in the Mammal Collection of the National Institute of Amazonian Research (INPA) in Manaus, Brazil (Supplementary Material).

**Figure 1:**
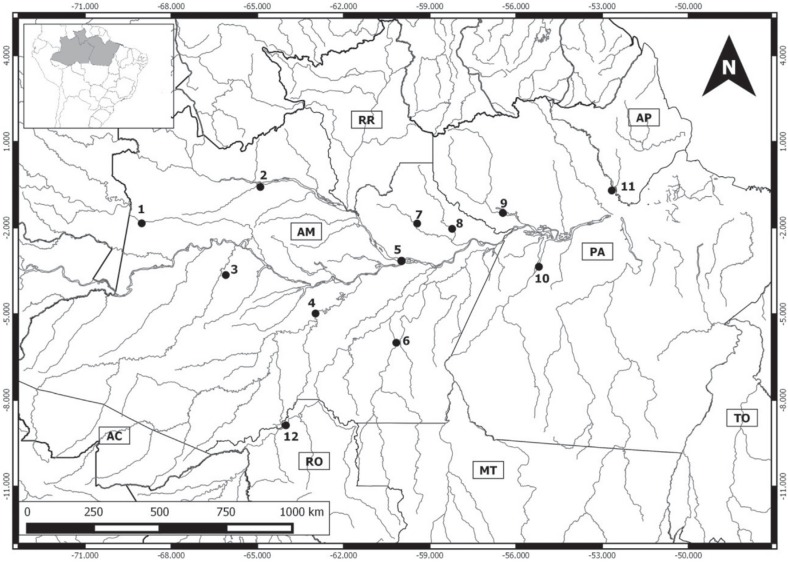
Collecting sites of marsupials; the locality refers to the municipality and the access river. Collecting localities: Brazilian state of Amazonas (AM): 1- Japurá River margins, municipality of Japurá (1.843416°S, 69.026472°W); 2- Negro River margins, Santa Isabel do Rio Negro (0.577250°S, 64.897694°W); 3- Juruá River margins, municipality of Juruá (3.641511°S, 66.100691°W); 4- Purus River margins, municipality of Tapauá (4.98066°S, 62.97700°W); 5- Negro River margins, municipality of Manaus (0.577250°S, 64.897694°W; 3.133333°S, 59.950000°W); 6- Aripuanã River margins, municipality of Novo Aripuanã (6.00000000000°S, 60.1666666667°W); 7- Uatumã River margins, municipality of Presidente Figueiredo (1.849988°S, 59.440200°W); 8- Jatapu River margins, municipality of São Sebastião do Uatumã (2.017940°S, 58.203228°W); Brazilian state of Pará (PA): 9- Trombetas River margins, municipality of Oriximiná (1.481638°S, 56.457333°W); 10- Tapajós River margins, municipalities of Aveiro and Santarém (3.35486111111°S, 55.2031666667°W); 11- Jari River margins, municipality of Monte Dourado (0.700000°S, 52.666666°W); Brazilian state of Rondônia (RO): 12- Madeira River margins, municipality of Porto Velho (8.87416°S, 64.007777°W). DATUM: WGS84.

**Table 1:** Species analyzed in the present study, and the code numbers correspond to the localities on the map (Figure 1).M= number of male individuals; F = number of female individuals; number of ITS = all counts recorded for the species at the respective locality.

Species	Locality number in Figure 1	M	F	Number of ITS per individual
*Caluromys philander*	10	3	1	0 (2)
	9	-	1	0
	5	-	1	0
	4	-	1	0
*Didelphis marsupialis*	5	1	1	0
*Glironia venusta*	12	-	1	0
*Gracilinanus* cf. *peruanus*	10	4	0	0
*Marmosa demerarae*	4	1	2	4, 6, 7
	10	-	3	6, 8, 12
	9	3	3	6, 7, 8
	11	1	-	7
	6	-	2	12
	3	1	-	10
*Marmosa murina*	7	1	1	2
	4	2	-	0
	6	-	1	0
	2	-	1	0
*Marmosops* cf. *pakaraimae*	1	-	1	12+XX
*Marmosops parvidens*	9	3	-	0, 2, 5, 12+X
*Marmosops pinheiroi*	10	1	-	12+X
	8	-	1	
	9	1	-	
*Metachirus nudicaudatus*	2	2	1	0
*Monodelphis* sp. nov.	4	1	-	2

The mitotic chromosomes were obtained in the field, from bone marrow, using the *in vivo* method described by Ford and Harmerton (1956). The comparative C-banding patterns were those described in Silva *et al.* (2017) that based their analyses on the technique described by Sumner (1972). Telomeric sequences were mapped using Fluorescent *in situ* Hybridization (FISH), as in Pinkel *et al.* (1986, with adaptations), with a stringency of 77%. The probes were obtained by PCR using standard primers for mammals, these being (TTAGGG)_5_ and(CCCTAA)_5_ (Ijdo *et al.*, 1991). The karyotypes were arranged based on the scheme of Patton (1967).

The C-banding patterns used for comparisons were those in Silva *et al.* (2017). The telomeric probe hybridized to the telomeric regions of both arms of all chromosomes in all 11 species (Fig. 2). However, six species, *Marmosops pinheiroi* (Pine, 1981), *Marmosops* cf. *pakaraimae* Voss, Lim, Díaz-Nieto & Jansa 2013, *Marmosops parvidens* (Tate, 1931), *Marmosa demerarae* (O. Thomas, 1905), *Marmosa murina* (Linnaeus, 1758) and *Monodelphis* sp., also presented telomeric sequences in the centromeric regions (ITS), which were co-located with the heterochromatin blocks (Figure 2).

**Figure 2:**
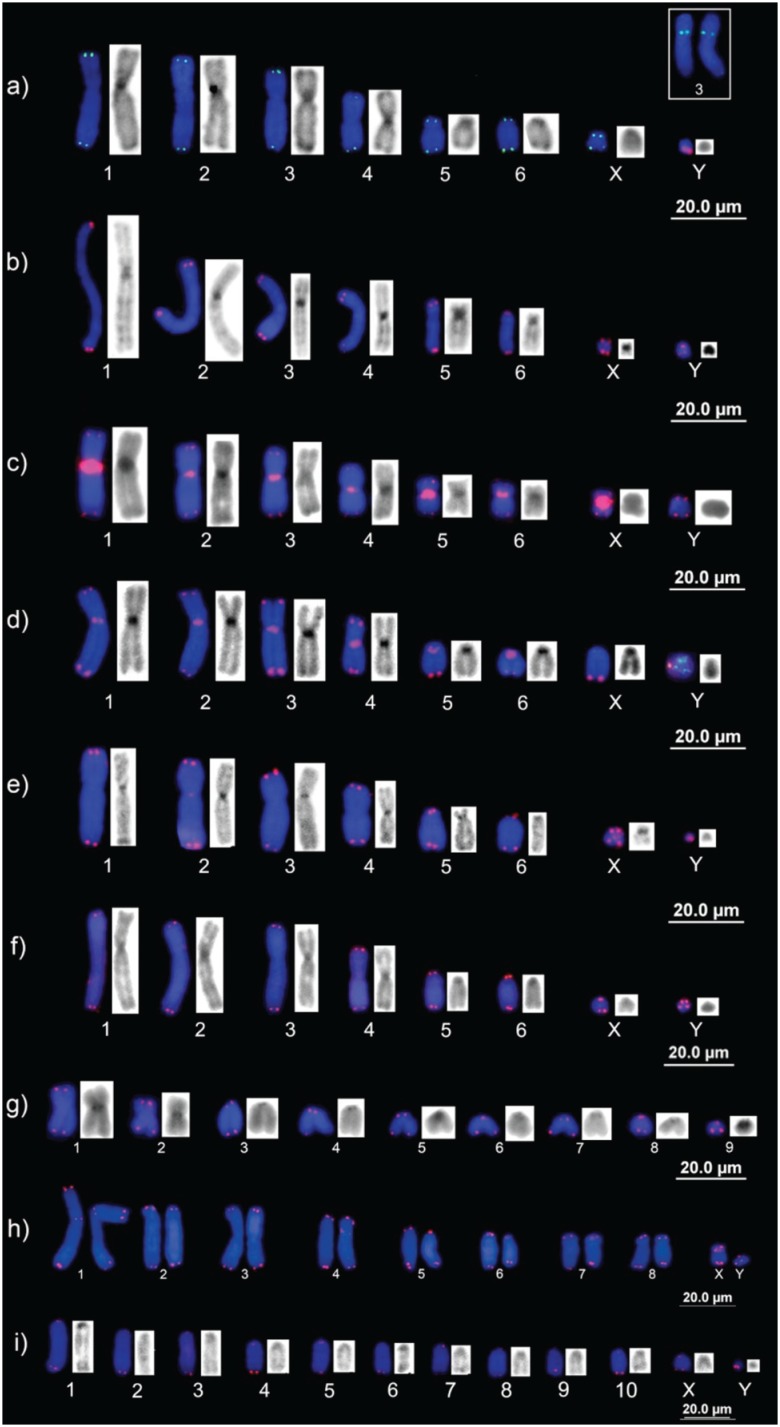
Collecting sites of marsupials; the locality refers to the municipality and the access river. Collecting localities: Brazilian state of Amazonas (AM): 1- Japurá River margins, municipality of Japurá (1.843416°S, 69.026472°W); 2- Negro River margins, Santa Isabel do Rio Negro (0.577250°S, 64.897694°W); 3- Juruá River margins, municipality of Juruá (3.641511°S, 66.100691°W); 4- Purus River margins, municipality of Tapauá (4.98066°S, 62.97700°W); 5- Negro River margins, municipality of Manaus (0.577250°S, 64.897694°W; 3.133333°S, 59.950000°W); 6- Aripuanã River margins, municipality of Novo Aripuanã (6.00000000000°S, 60.1666666667°W); 7- Uatumã River margins, municipality of Presidente Figueiredo (1.849988°S, 59.440200°W); 8- Jatapu River margins, municipality of São Sebastião do Uatumã (2.017940°S, 58.203228°W); Brazilian state of Pará (PA): 9- Trombetas River margins, municipality of Oriximiná (1.481638°S, 56.457333°W); 10- Tapajós River margins, municipalities of Aveiro and Santarém (3.35486111111°S, 55.2031666667°W); 11- Jari River margins, municipality of Monte Dourado (0.700000°S, 52.666666°W); Brazilian state of Rondônia (RO): 12- Madeira River margins, municipality of Porto Velho (8.87416°S, 64.007777°W). DATUM: WGS84.

*Marmosops pinheiroi* (Figure 2c) and *Marmosops* cf. *pakaraimae* (data not shown) were the species that had the largest number of chromosomes with centromeric ITS, including a conspicuous block in the centromere of the X chromosome. In *Marmosops parvidens* (data not shown), variation was found among individuals in the quantity of ITS, with, two, five, and 13 interstitial signals (Table 1). Inter-individual variation was also observed in *Marmosa demerarae*, which had up to 12 centromeric ITS, on all chromosomes except for the sexual ones (Figure 2d; Table 1). In *Marmosa murina*, the ITS were located in the centromere of the second autosomal pair and the X chromosome (Fig. 2e). In the case of *Caluromys philander* Linnaeus, 1758, a pericentromeric ITS was found in only one individual, collected on the Tapajós River region, in autosomal pair 3 (Figure 2a, box). *Monodelphis* sp. nov. presented a single ITS in the largest autosomal pair (Figure 2h). No ITS were detected in any of the other species, i.e., *Gracilinanus cf. peruanus* (O. Thomas, 1909), *Metachirus nudicaudatus* (Geoffroy-St.Hillaire, 1803), *Glironia venusta* O. Thomas, 1912 and *Didelphis marsupialis* Linnaeus, 1758 (Figure 2b, f, g, i). In *D. marsupialis*, the telomeric sites were much reduced in size in comparison with the other species (Figure 2i).

Six principal classes of Interstitial Telomeric Sequence (ITS) are recognized: heterochromatic (het-ITSs), short (s-ITSs), large ITSs in restricted euchromatic regions (Restricted eu-ITSs), long subtelomeric, fusion, and pericentromeric ones (Lin and Yan, 2008; Ruiz-Herrera *et al.*, 2008; Schmid and Steinlein, 2016).

We consider the ITS observed in the present study as being of the het-ITS type, in view of large blocks that coincide with the heterochromatin and vary in number. Metcalfe *et al.* (2004, 2007) recorded a similar scenario in Australian marsupials. If these sequences were in fact related to satellite DNA, rather than rearrangements, a number of questions arise. For example, what may have given rise to the DNA motifs in these regions? Additionally, how can the inter-individual variation observed in these species be accounted for?

Three principal mechanisms have been proposed to account for the origin of interstitial telomeric sequences: (i) insertions in double-strand breaks, which involve telomerase (Nergadze *et al.*, 2004, 2007), (ii) the fusion of the telomeric regions of chromosomes of different pairs (Bolzán and Bianchi, 2006), and (iii) transposition (Bouffler *et al.*, 1993; Nergadze *et al.*, 2007).

Insertion by repair mechanisms can be a cause. The incidence of solar radiation, in particular ultraviolet radiation has increased progressively and considering that didelphids fetuses complete their development outside the uterus, it may increase their exposure to radiation and provoke chromosomal breaks (Hartman, 1923; Petrides, 1949; Norval *et al.*, 2007). Telomerase actions could result in the formation of short ITS, subsequently repeated progressively (Messier *et al.*, 1996). Laboratory experiments have demonstrated the occurrence of mutations and chromosomal breaks in *Monodelphis domestica* (Pathak *et al.*, 1996). However, experiments need to be done to corroborate or refute such a hypothesis. Fusions can be another cause, and recurring points of chromosomal breakage have been identified in marsupials by chromosome painting and G-banding. Chromosomal fusions can be rejected *a priori* in the family Didelphidae (Pagnozzi *et al.*, 2002; Metcalfe *et al.*, 2004; Westerman *et al.*, 2010). In New World marsupials, chromosomal inversions appear to be more common in the process of chromosomal reorganization (Westerman *et al.*, 2010; Silva *et al.*, 2017). A third possibility is transposition, possibly intermediated by transposable elements (TEs), for example. Retrotransposition has been observed in cell lineages of the Chinese hamster, through retrotransposons of the LINE type (Nergadze *et al.*, 2007).

Irrespective of their origin, it is possible that the het-ITS arose as a short sequence, which was subsequently duplicated, increasing in length, and becoming integrated with the heterochromatin, making it detectable by FISH. This heterochromatinization would provide an alternative mechanism for the reduction of the risk of fissions or other rearrangements, and would account for the association between the ITS and the larger heterochromatin blocks, which are highlighted more intensely by C-banding. Whatever the exact process, it is important to recognize that these ITS arose and were fixed, and thus constitute part of the evolutionary history of the species, even though there is no evidence on their possible adaptive value.

Within the family Didelphidae, these ITS have been fixed, as far as it is known, only in species of the tribes Marmosini (*Marmosa* spp., *Monodelphis* spp.) and Thylamyini (*Marmosops* spp., *Gracilinanus microtarsus*) (Svartman and Vianna-Morgante, 1998; Carvalho and Mattevi, 2000; Pagnozzi *et al.*, 2002; present study), which did not recover a sister-group relationship (Voss and Jansa, 2009).

In *Marmosa murina* and *C. philander* (present study), the variation in the number of ITS was relatively discrete, and in fact, in *M. murina*, ITS have been observed only in Amazonian individuals, and not in those from other Brazilian regions (Carvalho and Mattevi, 2000; Pagnozzi *et al.*, 2002). In *C. philander*, the available records were restricted to the Amazonian individuals analyzed by Souza *et al.* (2013), and now, ITS were also detected in only one individual out of six analyzed in the present study.

Fixation of the ITS and their intraspecific variation appear to be the result of mechanisms that do not involve chromosomal fusion. This indicates that the ITS found in *Marmosops* spp., *Marmosa murina*, and *M. demerarae* are the result of the association between these sequences and the heterochromatin, specifically, with certain types of satellite DNA. However, the intraspecific variation found in the number of this marker is not directly related to a phylogenetic pattern, and it will only be possible to better understand the importance of these ITS by discovering the composition of these sequences as a first priority in determining their potential role, and to what extent they are conserved between species.

## Supplementary Material 1

The following online material is available for this article

Supplementary Material 1
