# Supplementary material for: Comparison of the heterochromatin and telomeric sequences distribuition in chromosomes of 11 species of Amazonian marsupials (Didelphimorphia; Didelphidae)
Source: Genet Mol Biol. 2020 May 11;43(2):e20190357. doi: 10.1590/1678-4685-GMB-2019-0357 (PMC7216969; doi:10.1590/1678-4685-GMB-2019-0357)
Supplement: Supplementary file 1 [file 1415-4757-GMB-43-2-e20190357-s1.pdf]

Supplementary Material to: Comparison of the heterochromatin and telomeric sequences distribuition in chromosomes of 11 species of Amazonian marsupials (Didelphimorphia; Didelphidae)

Supplementary Material 1

We deposited all individuals analyzed in the present study in the Mammal Collection of the National Institute of Amazonian Research (INPA) in Manaus, Brazil. The specimens are represented by their species name, collecting locality, and INPA's collection number and/or field number (within parentheses). **Voucher specimens: *Glironia venusta*:** (INPA4577 - BAC 80);

***Caluromys philander*:** Tapajós River (male: SISTAP-M 297; SISTAP-M 305; SISTAP-M 318; female: SISTAP-M 244); Trombetas River (female: SISTRO-M 652); Purus River (female: INPA 7330 - CAN 34); Negro River, Manaus (female: MSN 01);

Marmosops* cf. *pakaraimae: Japurá River (female: SISJAP-M 705);

Marmosops pinheiroi: Jatapu River (female: SISJAT-M 3); Trombetas River (male: SISTRO-M 600); Tapajós River (Male:SISTAP-M-237);

Marmosops parvidens: Trombetas River (male: SISTRO-M 501; SISTRO-M 532; SISTRO-M 533;)

Marmosa murina: Uatumã River (female: CEF 27; male: CEF 18); Negro River: (female: SISSIS-M 64; Purus River (male: INPA7339 - CAN 43; INPA 7346 - CAN 50); Aripuanã River (female: INPA 5385 - MCA12);

Gracilinanus* cf. *peruanus: Tapajós River: (male: SISTAP- M 245; SISTAP- M 343; SISTAP- M 344; SISTAP-M 345);

***Marmosa demerarae*:** Aripuanã River (female: INPA 5438 - MCA 65; INPA 5419 – MCA 46); Juruá River (male: EE 115); Tapajós River (female: SISTAP-M 241; SISTAP-M 369; SISTAP-M 321); Trombetas River (female:SISTRO-M 516; SISTRO-M 676 ; SISTRO-M 667; male: SISTRO-M 539; SISTRO-M 572; SISTRO-M 613); Purus River (female: INPA 7321 - CAN 25; INPA 7327 - CAN 31; male: SISPUR-M 149); Jari River (Female: RNL31).

***Monodelphis* sp. nov.:** Purus River: (male: INPA 7340 - CAN 44);

***Metachirus nudicaudatus*:** Negro River: (female: SISSIS-M 78; male: SISSIS-M 84; SISSIS-M 116);

Didelphis marsupialis: Manaus: (female: EE 204; male: EE 206).

Figure 2: karyotyped Specimens: a) *Caluromys philander* (SISTAP-M 244, box: SISTAP-M 297, Y chromosome (SISTAP-M 305); b) *Gracilinanus* cf. *peruanus* (SISTAP-M 344); c) *Marmosops pinheiroi* (SISTAP-M 237); d) *Marmosa demerarae* (INPA 5438 - MCA 65), e) *Marmosa murina* (CEF 18); f) *Metachirus nudicaudatus* (SISTAP-M 302); g) *Glironia venusta* (INPA 4577 - BAC 80); h) *Monodelphis* sp. nov. (INPA 7340 - CAN 44); i) *Didelphis marsupialis* (EE 206).
